# Can microbes compete with cows for sustainable protein production - A feasibility study on high quality protein

**DOI:** 10.1038/srep36421

**Published:** 2016-11-08

**Authors:** Mike Vestergaard, Siu Hung Joshua Chan, Peter Ruhdal Jensen

**Affiliations:** 1 Department of Microbial Biotechnology & Bio refining, Technical University of Denmark, Lyngby, Denmark

## Abstract

An increasing population and their increased demand for high-protein diets will require dramatic changes in the food industry, as limited resources and environmental issues will make animal derived foods and proteins, gradually more unsustainable to produce. To explore alternatives to animal derived proteins, an economic model was built around the genome-scale metabolic network of *E. coli* to study the feasibility of recombinant protein production as a food source. Using a novel model, we predicted which microbial production strategies are optimal for economic return, by capturing the tradeoff between the market prices of substrates, product output and the efficiency of microbial production. A case study with the food protein, Bovine Alpha Lactalbumin was made to evaluate the upstream economic feasibilities. Simulations with different substrate profiles at maximum productivity were used to explore the feasibility of recombinant Bovine Alpha Lactalbumin production coupled with market prices of utilized materials. We found that recombinant protein production could be a feasible food source and an alternative to traditional sources.

The global population is estimated to reach 9.1–9.7 billion by 2050, with approximately an 1.8-fold increase in income per capita^1^,^2^. Food consumption is predicted to become increasingly based on animal derived products – mainly due to increased wealth in developing countries^2^. The production of valued protein rich foods, such as milk is estimated to increase by 62% (from production levels of 2005–2007 to 2050, i.e. from 664 to 1 077 million tons)^2^. Animal derived products are less sustainable to produce compared to their vegetable counterparts. This is why it is important to either produce products that can be used as an alternative or develop better ways of producing animal derived products. Certain subcomponents derived from milk, especially proteins, are important due to their functionality and nutritional value, which are why technologies that can produce these more sustainably would be beneficial. Recombinant protein production could provide an alternative source of milk proteins, as engineered microorganisms have the advantage of producing specific compounds in elevated amounts^3^. This approach has revolutionized the biotechnology and pharma industry^4^,^5^ though proteins for human consumption have not yet been subject to such trends, as competition from traditional industries and GMO controversies have been of concern. Proteins used as ingredients in the food industry have traditionally been based on isolates from natural sources – with milk proteins as a major driver^6^. The nutritional and functional capacities of certain milk proteins make them an obvious choice when fortifying or formulating products and the production of milk proteins by recombinant means could offer a sustainable additional source in the future. It is however nontrivial to evaluate the economic feasibility of such an endeavor, as cost of substrate, price of milk proteins and technical performance becomes decisive.

The Genome-Scale Metabolic Network (GSMN) is the collective knowledge of metabolic routes within a given organism, while Flux Balance Analysis (FBA) is the simulation within these networks that allows metabolites to change accordingly^7^. The sandbox like nature of the GSMN makes it possible to use it as a tool for simulating metabolic conversions and growth. Here we made a model where the upstream feasibility of recombinant protein production was explored within the GSMN of the *E. coli* (iAF1260)^8^ for the milk protein, Bovine Alpha Lactalbumin (α-La) (Uniprot: P00711). Briefly, this was achieved by associating the required metabolites for α-La production with costs and dynamically simulating the optimal production with respect to different objective functions, i.e. maximizing productivity using sugar and soybean meal as substrates. Simulations within the model were assessed and evaluated in terms of economic potential. The results indicated a potential for recombinant food protein production in the future.

The dairy industry has within the last decades been revolutionized with respect to processing and purifying proteins from milk and whey. Proteins can be isolated from whey, a side product generated during cheese manufacturing, which historically has been a waste product. Revenue generated from protein extraction has been increasing in recent decades and has comprised a growing part of the total revenue of the dairy industry. The market for whey proteins has seen a predominant growth within whey protein isolates (WPI – above 90% protein purity) compared to lesser whey protein concentrates (WPC34–34% protein purity) within the last decade, implying that pure proteins with functional properties are in high demand. The total production of WPI was 36.800 mt in 2014 for the US, which is an increase of 2.5 fold compared to the total production in 2006–14.400 mt (http://usda.mannlib.cornell.edu/MannUsda/viewDocumentInfo.do?documentID=1052). WPI’s or fractions of these are produced by a variety of filtration systems and chromatographies^6^. Among the products formulated with whey proteins are infant formulas, where especially the Bovine α-La is used to mimic the content of Humane α-La found in human milk due to its beneficial properties^6^,^9^. α-La is sold at different purities in powder form - ranging from 35% to 65% α-La purity in 80–90% total protein content. The price range lies approximately between 15 €/kg to 25 €/kg according to purity as of 2014 (Inquiry at international α-La vendor).

## Results

The conversion of lower valued carbon sources into higher valued food proteins by microbial means could become a sustainable contribution in the future and it is therefore relevant to explore its economic feasibility. The created model utilizes the *E. coli* (iAF1260) base model and adds the option to shift a part of the metabolites designated for growth into recombinant protein production. This is achieved by adding, uptake rates of relevant metabolites, metabolic cost of protein production and ribosomal elongation rates, while the feasibility is evaluated by assigning cost to substrate materials and gain from produced α-La - as described in the method section. Process simulations were obtained by exhaustively maximizing productivity within the hypothetical outcome of cell growth and recombinant protein production at different substrate compositions. The results from the simulations are depictured as 3 dimensional surface planes where the individual coordinates represent different simulated processes with the global maximum being the best process. The model does not impose any time requirements and the different processes vary in time – as described in the method section.

### Productivity of α-La

**Figure 1:**
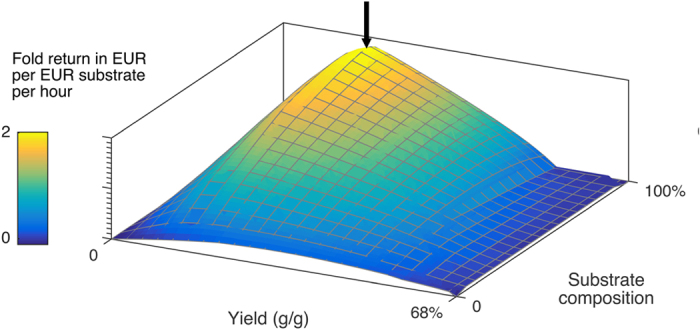
Simulation of economic productivity. The plot depictures α-La fermentations at maximized productivity with yield per substrate, substrate compositions and economic return based on the substrate price per hour on the X, Y and Z axis respectively. The global maximum represents a fermentation, which converts the initial substrate into α-La worth 21.8 times the value of the starting material. The fermentation is simulated to take 11 hours and require the substrate to compose of 5% soybean and 95% sugar.

Results from the simulations where productivity were optimized are presented in Fig. 1 as a plot with yield per substrate, substrate compositions and productivity on the X, Y and Z axis respectively. A color layer representing the economic return based on the substrate price is furthermore applied and utilizes a per hour unit in order to compare simulations with different process times. The plot shows that the highest productivity occurs at a process with an average of 0.2 g produced protein per gram substrate (20%) with a substrate composition of 95% sugar and 5% soybean – indicating that some amino acid supplements in the form of soybean meal are favorable compared to media based on sugar exclusively. This demonstrates that a more expensive and less energy dense substrate like soybean meal can be applicable, as a greater flux of metabolites matters when maximizing productivity. The upstream process takes 11 hours and converts the initial substrate into α-La worth 21.8 times the value of the starting material. The underlying growth profile for the process is exponential with a specific growth rate that starts of as a plateau at a high rate, then declines over the course of the process and ends at a plateau of lower specific growth rate towards the end. The substrates are fully utilized during the process (which is not the case for all the simulations) and the ribosomes are at maximum capacity throughout the simulations. See Supporting Data - 5 for supporting figures. The economic return in the model is derived from the market price of 60% pure α-La, which includes all related costs e.g. expenditures for purification and distribution. The simulations potential economic return is thereby lower as additional cost needs to be applied to account for the further processing.

**Figure 2:**
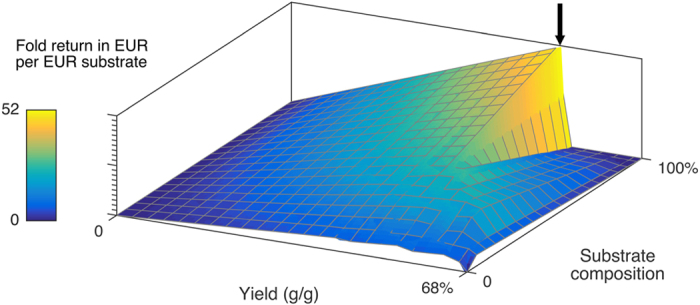
Simulation of economic yield. The plot depictures α-La fermentations at maximized productivity with yield per substrate, substrate compositions and economic return based on the substrate price on the X, Y and Z axis respectively. The global maximum represents a fermentation, which converts the initial substrate into α-La worth 52 times the value of the starting material. The fermentation is simulated to take 300 hours and require the substrate to compose of 100% sugar.

**Figure 3:**
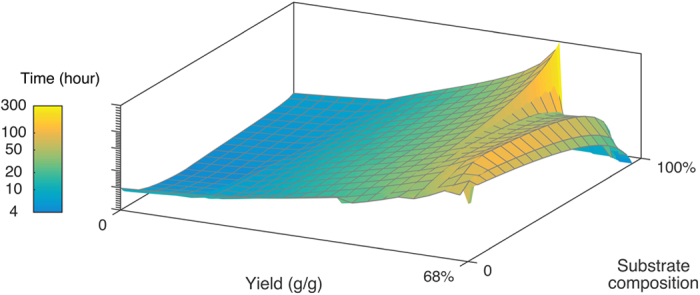
Total fermentation time. The plot depictures α-La fermentations at maximized productivity with yield per substrate, substrate compositions and time on the X, Y and Z axis respectively. The simulations vary greatly in total time, from a few hours to almost two weeks. Simulations with high yield per substrate do in general take longer. The rapid decline along the 60% yield (g/g) simulations seen in Figs 2 and 3 is a result of the cell growth and recombinant protein production being unable to maintain suitable productivity for prolonged fermentations.

Maximizing productivity provides simulated processes where the balance between biomass and protein production are shifted towards biomass, as increased biomass facilitates larger fluxes of protein over time compared to yield maximized processes. For situations where time is less of a concern and process yield is more important, the shift towards protein production can be utilized to increase the feasibility per cost of substrate. By recognizing that productivity always will be a desirable process parameter, the surface plane of the productivity plot was analyzed and a plot with the final process yield was created based on the productivity simulations. The plot is presented in Fig. 2 with yield per substrate, substrate compositions and process yield on the X, Y and Z axis respectively. The color over layer represents the economic return based on the substrate price. The plot demonstrates that higher process yields can be obtained at simulations with higher average produced α-La per gram substrate. The maximum process yield is present in a process with a 52 fold economic return per substrate at 0.5 gram produced protein per gram substrate at 100% sugar media composition. The process takes approximately 300 hours and is therefore likely to be associated with costs that would decrease the actual gain of running the process (labor and overhead) and technical issues (protein stability and cell death). Figure 3 presents the total fermentation time utilized in the simulation of economic productivity (Fig. 1) and simulation of economic yield (Fig. 2).

The process yield and the productivity represent two bases of the production strategy, namely cost-efficiency and time-efficiency. The solutions existing in between are compromises and can be applied for complex processes where neither of the two parameters is completely dominating the economic feasibility. A combination of the two objectives can be maximized with the parameter *α* depending on the demand or growth of the market, see equation 1.


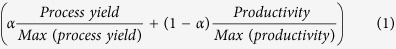


where 0 < α < 1.

The simulated upstream production of α-La demonstrates the potential of recombinant protein production in microorganisms and the cellular machinery that supports it. Processes based on microorganisms could become an additional food source and is worth considering when discussing the possible solutions for future supply of high quality proteins for human consumption^10^. The further downstream process of a theoretical α-La production would determine the overall feasibility, as purification costs can be significant in biotech processes.

### Processing

*E. coli* is a prokaryote industrial platform which has been widely used for recombinant protein production in biotech and pharmaceutical applications and could be a candidate for recombinant food protein production. While there are a variety of different technical obstacles that makes recombinant protein production challenging, it is important to recognize that the underlying biological system is capable of converting biomass into specific products at a rapid pace, which could be utilized to secure adequate amounts of quality food proteins. Different strategies employed in recombinant protein production enable streamlined downstream processes and recombinant products can in general be rendered available for processing by multiple initial strategies, including intracellular recovery, extracellular secretion or intracellular recovery from inclusion bodies^11^. The cost of purification is a significant factor that affects the overall feasibility, though the current market of GMO food graded vitamins, preservatives and enzymes suggests that cost effective processes are available.

The cost of handling and terminating GMO material is an additional expenditure for any recombinant process, though the cost can be mitigated by recycling the microbial biomass produced. One approach is to seek authorization to use specific terminated GMO as feed products. Terminated GMOs used as feed material for animals can create value associated with sales and reduced cost in waste management, providing a sustainable alternative. Insulin producer Novo Nordisk and amino acid producer Ajinomoto Eurolysine SAS are examples of companies with different types of end-products which have sought such an authorization for some of their terminated GMO by-products (http://ec.europa.eu/food/dyna/gm_register/index_en.cfm).

### Legislative concerns

The use of GMO derived ingredients intended for human consumption is under legislative control. Approval from governmental authorities is granted if sufficient documentation is presented in regards to production methodology and consumer safety. The probability of achieving usage authorization for α-La isolates from *E. coli* can be evaluated based on prior cases. The US FDA GRAS Notice Inventory, lists food ingredients that are regarded as safe to use and includes protein isolates from milk and whey (See GRN No. 37 and GRN No. 444 (http://www.accessdata.fda.gov/scripts/fdcc/). *E. coli* strain K-12 is among other used in the production of the recombinant enzyme, Chymosin, applied in cheese manufacturing. Chymosin is regarded as safe to use, when produced in certain non-pathogen microorganisms, including *E. coli* strain K-12 - according to § 184.1685 (http://www.gpo.gov/fdsys/pkg/CFR-2012-title21-vol3/pdf/CFR-2012-title21-vol3-sec184-1685.pdf). Chymosin is an example of a food ingredient that is dominating the market, as the natural source is not available in sufficient amounts nor at attractive prices. For theoretical α-La protein isolates from *E. coli* K-12, both compound and organism have been approved separately in prior cases for food formulation, which indicate that authorization could be obtained.

### Production

The formation of amino acids followed by their subsequent polymerization into proteins is a notable expenditure in metabolites, as it requires both substrate derived building blocks and energy in the form of ATP^12^,^13^. The increased cost of amino acid compared to sugar does not decrease the feasibility of using amino acids in the model, as higher amino acid fluxes results in higher productivity of the recombinant protein. The capacity of the TCA cycle has been reported as a limiting factor during recombinant protein production in *E. coli* on minimal medium as the formation of intermediates for amino acids and intermediates designated for oxidative phosphorylation competes for substrates^14^. Media composition is an important parameter for industrial processes. The shift between protein production and growth is only limited in the model by the metabolites required for mRNA and subsequently protein synthesis, which implies two things: (1) the translation initiation of recombinant protein is not limited by physical constraints and (2) the utilized strain is capable of mimicking the required production profile through a controllable system. Elaborate algorithms and models exist that can overcome limitation at translation initiation by rational design^15^,^16^, while several controllable systems for protein production are available in bacteria at industrial scale^17^. Protein translation involves four phases: initiation, elongation, termination and ribosome turnover, where initiation usually is the limiting factor in recombinant production^11^,^16^ implying that the applied genetic material is limiting its own protein synthesis (inadequate transcription levels, mRNA instability, unfavorable ribosomal-binding-site complex). In the case where initiation limitations are overcome, the native genetic blueprint of the organism becomes the limiting factor for recombinant protein synthesis. In such cases the protein elongation rate in conjunction with the amount of ribosomes becomes the limiting rate - as detailed in the model. *In vivo* studies additionally suggest that the maximum elongation rate per ribosome is higher than the experimental observed^18^.

### Perspective

The data used in the simulations are sensitive to fluctuations in cost of substrates and protein prices. A sensitivity analysis could be incorporated to predict feasibility based on projected trends in the market. To compete with traditional production the conversion of input material into α-La needs to be economically feasible, though the initial cost associated with acquiring production capacities at large scale, developing strain(s) and acquiring legislative approval, determines if it is an attractive investment. Alternative production methods utilizing recombinant protein production could become an important source for ingredients that would otherwise become scarce in the future or unsustainable to produce. A food industry integrated with recombinant production capabilities would be less susceptible to traditional negative events and could create novel products not currently available. Recombinant α-La production and other milk proteins could be incorporated into the dairy industry by utilizing their existing pipeline and infrastructure, while strains able to utilize the available material in the dairy industry could be further integrated into the process.

Bovine α-La is used as a substitution to Human α-La in infant formulas, as there are no feasible sources of Human α-La. Recombinant human α-La production could be envisioned, though controversies could arise due to the human origin. The controversy surrounding the use of GMOs for food production needs to be addressed and superior products, both in terms of quality and cost could help alleviate the negative image surrounding recombinant proteins for food. This in addition to rigorous safety tests would be needed to make recombinant products become marketable.

### Assumptions and limitations

The data used for substrate uptake and protein chain elongation rate are from different *E. coli* strains obtained at slightly different experimental setups than that of the base model. We estimated the data to be sufficiently accurate to describe and limit protein production in conjunction with the GSMN, and to evaluate the initial feasibility of recombinant α-La production. The model does furthermore assume that the only burden imposed on the cells is the metabolic cost derived from the recombinant production, which is a simplification. Effects such as, ATP-dependent proteolytic degradation by the proteasome, increased chaperone requirements or protein instability are therefore neglected^19^. The biomass accumulation and the derived volume for optimal growth have not been taken into consideration and different fermentation modes have not been explored. The price of α-La is derived from its purity and functional properties and the model assumes that these properties are kept.

## Discussion

The production of food proteins by GMO organisms could be one solution to an increasing population with a demand for quality proteins and sustainable production methods, though such solutions are challenging to evaluate economically. It is therefore important in an industrial context to have models that can translate biological data and processes into economic values and thereby evaluate different innovative ideas. The GSMN system allows simulations of growth and product production, which with an emphasis on economic feasibility and process parameters can be used as an evaluation tool for industrial processes. Some works exist that cover certain requirements for the industry^20^,^21^,^22^ though little has been done to bridge the economic and metabolic processes relevant for recombinant production. In this study, we created a novel model, to evaluate the production of recombinant α-La in *E. coli* by utilizing ribosomal data in conjunction with the base model iAF1260. We found the conversion of substrate into product could result in approximately 22 fold economic return in a rapid upstream process at maximized productivity, which potentially could provide a sufficient income for the feasibility of an overall process. It is further possible to increase the cost efficiency by prolonging the process and obtaining more α-La by utilizing more time. With the model as the metabolic basis it is possible to build integrated economic models based on markets and processing inputs and study their performance. We hope that this kind of interdisciplinary approach can evaluate recombinant ways of producing products and support a sustainable translation into a bio based economy.

## Methods

### Protein synthesis general

The potential feasibility of recombinant α-La production was assessed in the framework of GSMNs by assigning economic values to input materials and output α-La. Commercial available sugar and soybean meal, reported by the World Bank, at prices of 0.41 €/kg and 0.50 €/kg respectively for Europe in 2014 (Dollar to euro = 0.95)^23^ were used as input material. For simplicity, the sugar and soybean meal were assumed to be in their monomeric forms: glucose, fructose and amino acids, while no other compounds were accounted for in the simulations. The respective product specifications can be seen in Supporting Data 1. The maximum uptake rates for amino acids were set according to the exponential growth data presented^24^ for *E. coli* and the glucose and oxygen maximum uptake rates were set accordingly^8^. The maximum uptake rate of fructose was arbitrarily set to that of glucose and defined, as an uptake that only occurs after glucose depletion. See Supporting Data 2 for a complete list of uptake rates. The biochemical reaction of protein production was written in terms of the metabolites in the GSMN with the stoichiometry for amino acids calculated from the coding sequence of α-La and the ATP cost estimated from protein synthesis and RNA transcription. Polymerization of amino acids into protein was set to a metabolic cost of 4.5 ATP per amino acid, reflecting aminoacyl-tRNA synthesis (ATP to AMP) and peptide bond formation at ribosomal level (2GTP to 2GDP) and an additional 0.5 ATP for miscellaneous quality processes^12^,^25^. mRNA synthesis was set to the metabolic cost of mRNA elongation (2.0 ATP)^12^, with 20 translations per mRNA molecule^26^ The synthesis of mRNA building blocks was neglected, as reuse occurs. A total of 41.6 mmol ATP per g α-La were used in the simulations. See Supporting Data 3 for further information. The synthesis rate of protein was coupled to the specific protein elongation rate at different cellular growth rates for *E. coli* per cell mass and ribosome content as reviewed in ref. 26. See Supporting Data 4 for further explanation and information on precise implementation. The market price of 25 €/kg for α-La extracts purified to the extent of 60% purity was used to generate a corrected theoretical price of 41,7 €/kg α-La. The database BioNumbers was used to find valuable information^27^.

### Optimization model for predicting optimal medium composition and protein yield -Bilevel dynamic optimization model

The reaction was added to network by inserting the column vector of stoichiometry into the stoichiometric matrix **N**. The capability of the production strain to utilize different substrates is encoded in **N** and constrained by the maximum substrate uptake rates. Given this capability, a bilevel dynamic optimization model was developed to predict the optimal medium composition and the protein yield to be achieved such that the economic return and the rate of return would be maximized:


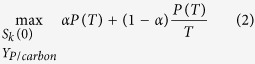



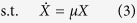



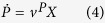



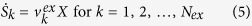



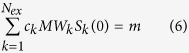



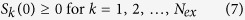



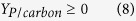



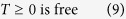



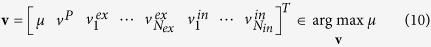



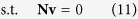



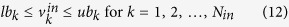



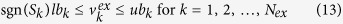



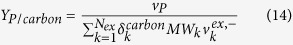


In the above model, the initial amounts of the *N*_*ex*_ extracellular substrates *S*_*k*_(0) for *k* = 1, 2, …, *N*_*ex*_ (7) and the protein yield on carbon source Y_*P*/carbon_ (8) are the constant control variables of the outer-level decision. The terminal time *T* is not fixed (9) so that the optimal time for stopping a batch would also be determined. The inner-level variable is the flux distribution 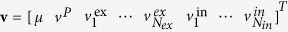 where *μ* is the specific growth rate (h^−1^), approximated by the biomass production flux in the GSMN, *v*^*P*^ the specific production rate of protein(g gdw^−1^ h^−1^), 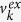 the exchange flux of the *k*-th extracellular metabolite (mmol gdw^−1^ h^−1^) and 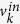 the flux of the *k*-th intracellular reaction (mmol gdw^−1^ h^−1^) assuming there are *N*_*in*_ intracellular reactions. The convention of uptake flux being negative and secretion flux being positive for 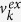 was adopted.

### Flux Balance Analysis (FBA) with protein yield constraint as the Inner-level problem

The flux distribution **v** is subject to the optimality criterion of maximum growth rate (10), the constraint of mass balance (11), upper bounds (*ub*_*k*_) and lower bounds (*lb*_*k*_) for intracellular fluxes (12) and exchange fluxes (13) respectively. sgn(*S*_*k*_) is the sign function for *S*_*k*_ so that when substrate *k* is used up, i.e. *S*_*k*_ = 0 then 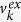 ≥ 0 and substrate *k* cannot be consumed anymore in the GSMN. Constraints (10–13) are the standard flux balance analysis FBA. An additional protein yield constraint (14), where 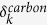 = 1 if substrate k is a carbon source or 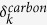 = 0 otherwise; *MW*_*k*_ is the molecular weight (g mmol^−1^) of substrate *k*; and 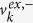 is the negative part of the flux, i.e. 
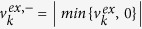
. This constraint ensures that a fraction of the carbon source taken up (in gram) is converted into the target protein. Constraints (10–14) collectively form the inner-level problem. Given the substrate availability *S*_*k*_ and the required protein yield *Y*_*P*/carbon_, a steady-state flux distribution maximizing growth rate could be determined.

### Dynamic Flux Balance Analysis (dFBA) and cost constraint as the outer-level problem

Constraints (3–5) are the state equations that describe the rate of change of biomass *X* (in gdw), protein amount *P* (in gram) and extracellular metabolite *S*_k_ (in mmol) respectively. These constraints combined with the inner-level problem (10, 11–14) is equivalent to dynamic flux balance analysis (dFBA)^28^,^29^. To optimize the economic return of the fermentation process described by dFBA, the total cost of the initial substrate amounts *S*_k_(0) is fixed to be *m* (in any currency) in constraint (6) where *c*_k_ is the cost of one gram of substrate *k*. With the cost fixed, the outer-level objective function (2) maximizes a convex combination of the protein produced *P*(*T*) and the production rate *P*(*T*)/*T*. This equivalently maximizes the economic return (price/gram)x *P*(*T*)/*m* and the rate of return (price/gram)x *P*(*T*)/*T*/*m*.

### Solution technique

The presented optimization model is dynamic, non-linear and bilevel and thus very difficult to solve in general. In the particular case of two available substrates, however, there are only two outer-level decision variables, namely the yield and the amount of substrate 1, because the amount of substrate 2 would be fixed by constraint (6). Note that both the yield and substrate amount are bounded below and above respectively. In this case, the problem can be solved by exhaustive search through the whole 2-dimensional solution space. Given the substrate amount and the yield, dFBA is performed and the optimal time allowing the maximum objective function value and the corresponding state variables are taken as a solution.

The model is concentration free and operates with grams and moles. The simulations have been done with input material equal to 0.004 € which is approximately 10 grams of substrate - depending on the substrate composition. The initial biomass is 0.01 g for the simulations and gas and adequate stirring is presumed.

## Additional Information

**How to cite this article**: Vestergaard, m. *et al*. Can microbes compete with cows for sustainable protein production - A feasibility study on high quality protein. *Sci. Rep.* **6**, 36421; doi: 10.1038/srep36421 (2016).

**Publisher’s note**: Springer Nature remains neutral with regard to jurisdictional claims in published maps and institutional affiliations.

## Supplementary Material

Supplementary Information
