# Supplementary material for: Can microbes compete with cows for sustainable protein production - A feasibility study on high quality protein
Source: Sci Rep. 2016 Nov 8;6:36421. doi: 10.1038/srep36421 (PMC5099699; doi:10.1038/srep36421)
Supplement: Supplementary Information [file srep36421-s1.pdf]

Supplementary data: Can microbes compete with cows for sustainable protein production - A feasibility study on high quality protein

Mike Vestergaard, Siu Hung Joshua Chan and Peter Ruhdal Jensen

Supporting Data – 1

Commercial available sugar and soybean meal, reported by the Worldbank, at prices of 0.41 €/kg and 0.50 €/kg respectively for Europe in 2014 (Dollar to euro = 0.95) where used as inputs material. The sugar and soybean meal where assumed to be in their monomeric form; glucose and fructose and amino acids - no other compounds were accounted for in the simulations. The respective product specifications can be seen below. The market price of 25 €/kg for α-La extracts purified to the extent of approximately 60% purity was used to generate a corrected price for the model: 41,7 €/kg α-La. This implies that the product has to be purified to the extent of 60% for the models output to be valid. Increased or decreased purity will impact the price accordingly.

Raw Sugar:

Sucrose (342.39 g/mol) at 0.41 €/kg contains in term of metabolites inserted into the dFBA assuming 97.5% purity per kilo:

Glucose 180.16 g/mol *0.5*0.975 = 2.71 mol glucose/kg
Fructose 180.16 g/mol *0.5*0.975 = 2.71 mol fructose/kg
Resulting in 0.08 €/mol Sucrose

Reference:
(http://go.worldbank.org/4ROCCIEQ50) - HISTORICAL DATA (XLS) Annual.

Soybean meal

Soybean meal at 0.50 €/kg contains 44% protein with the following amino acid composition:

Amino Acids	%	MW	mmol per kg soybean meal
Ala (A)	2.52	71.09	354.48
Arg (R)	3.4	156.2	217.67
Asn (N)	2.52	114.12	220.82
Asp (D)	2.52	115.1	218.94
Cys (C)	0.67	103.16	64.95
Gln (Q)	2.52	128.15	196.64
Glu (E)	2.52	129.13	195.15
Gly (G)	2.52	57.07	441.56
His (H)	1.1	137.16	80.20
Ile (I)	2.5	113.18	220.89
Leu (L)	3.4	113.18	300.41
Lys (K)	2.7	128.19	210.62
Met (M)	0.65	131.21	49.54
Phe (F)	2.2	147.19	149.47
Pro (P)	2.52	97.13	259.45
Ser (S)	2.52	87.09	289.36
Thr (T)	1.7	101.12	168.12
Trp (W)	0.6	186.23	32.22

Tyr (Y)	2.52	163.19	154.42
Val (V)	2.4	99.15	242.06

Reference:

(http://go.worldbank.org/4ROCCIEQ50) - HISTORICAL DATA (XLS) Annual.

(http://www.soymeal.org/composition.html at the bottom of the page) The percentages of the following amino acids are defined (in red) while the remaining are set as an arbitrary average (in black)

Glucose syrup

Glucose syrup (180.16 g/mol) at 0.75 €/kg in 2014 (in dry weight) contains in term of metabolites inserted into the dFBA assuming 97.5% purity per kilo:

Glucose 180.16 g/mol *0.975 = 5.42 mol glucose/kg

Resulting in 0.14 €/mol glucose

Reference:

(http://ers.usda.gov/data-products/sugar-and-sweeteners-yearbook-tables.aspx) 'Table 7--U.S. wholesale list price for glucose syrup, Midwest markets, monthly, quarterly, and by calendar and fiscal year 1; (Dollar to euro = 0.95).

Supporting Data - 2

The maximum uptake rates for amino acids were set according to the exponential growth data presented in Selvarasu et.al, while the glucose and oxygen maximum uptake rates were set according to Feist et al. The maximum uptake rate of fructose was arbitrarily set to that of glucose and defined, as an uptake that only occur after glucose depletion.

Substrate	Maximum uptake rate in (mmol/gdw/h)
Oxygen	18.5
Glucose	8
Fructose	8
Ala (A)	0.718
Arg (R)	2.28
Asn (N)	0.08
Asp (D)	0.395
Cys (C)	0
Gln (Q)	0
Glu (E)	0.868
Gly (G)	0.027
His (H)	0
Ile (I)	0.173
Leu (L)	0.457
Lys (K)	0.187
Met (M)	0.163
Phe (F)	0.188
Pro (P)	0.187
Ser (S)	3.355
Thr (T)	0.562
Trp (W)	0.096
Tyr (Y)	0
Val (V)	0.139

Reference:

Selvarasu S, Ow DSW, Lee SY, et al. Characterizing Escherichia coli DH5α growth and metabolism in a complex medium using genome-scale flux analysis. Biotechnol Bioeng. 2009;102(3):923-934. doi:10.1002/bit.22119.

Feist AM, Henry CS, Reed JL, et al. A genome-scale metabolic reconstruction for Escherichia coli K-12 MG1655 that accounts for 1260 ORFs and thermodynamic information. Mol Syst Biol. 2007;3(121):121. doi:10.1038/msb4100155.

Supporting Data - 3

The biochemical reaction of protein production was written in terms of the metabolites in the GSMN with the stoichiometry for amino acids calculated from the coding sequence of the protein and the ATP cost estimated from the protein synthesis and RNA transcription. Polymerization of amino acids into protein was set to a metabolic cost of 4.5 ATP per amino acid, while mRNA elongation was set to a metabolic cost of 2.0 ATP with 20 translations per mRNA molecule. A total of 41.6 mmol ATP per g α-La were used in the simulations.

Calculation

Amino acids:	Times accruing:	MW* (g/mol):	Molar fraction (of 1 mol protein)	mmol AA for 1 g of protein
Ala (A)	3	71,09	2,42%	0,210
Arg (R)	1	156,2	0,81%	0,070
Asn (N)	8	114,12	6,45%	0,559
Asp (D)	13	115,1	10,48%	0,909
Cys (C)	8	103,16	6,45%	0,559
Gln (Q)	6	128,15	4,84%	0,420
Glu (E)	7	129,13	5,65%	0,489
Gly (G)	6	57,07	4,84%	0,420
His (H)	3	137,16	2,42%	0,210
Ile (I)	8	113,18	6,45%	0,559
Leu (L)	13	113,18	10,48%	0,909
Lys (K)	12	128,19	9,68%	0,839
Met (M)	2	131,21	1,61%	0,140
Phe (F)	4	147,19	3,23%	0,280
Pro (P)	2	97,13	1,61%	0,140
Ser (S)	7	87,09	5,65%	0,489
Thr (T)	7	101,12	5,65%	0,489
Trp (W)	4	186,23	3,23%	0,280
Tyr (Y)	4	163,19	3,23%	0,280
Val (V)	6	99,15	4,84%	0,420
Total including new Met(M):	124	Including new Met(M)		8,671

Reference/tools: ***http://www.uniprot.org/uniprot/P00711*** ***http://web.expasy.org/protparam/***

**ATP - amino acid polymerization cost**	4,5	ATP per amino acid	**39,01751**	mmol ATP per g protein
**ATP - mRNA elongation cost**	2	ATP per nucleotide	**2,601168**	mmol ATP per g protein
Total number of nucleotides for mRNA:	372			
Translations per mRNA:	20			
**Total ATP cost:**			**41,61868**	mmol ATP per g protein

Reference:

A theoretical study on the amount of ATP required for synthesis of microbial cell material. A. H. Stouthamer. 1973.
The McCree-de Wit-Penning de Vries-Thornley Respiration Paradigms: 30 Years Later. J.S Amthor. 2000.

Supporting Data - 4

The synthesis of protein was incorporated into the model based on the specific protein elongation rate at different cellular growth rates for E.coli per cell mass and ribosome content and then further constrained by the amount of endogenous protein production.

[AA elongated for biomass (mmol/gdw) * growth rate (/h)] + [AA elongated for BAL (mmol/gdw protein) * production rate (gdw protien/gdw biomass/h)] ≤ maximum elongation rate while the maximum elongation rate = m * growth rate + c.

	Doubling time (Min)					
	t,100 - ∞	t,60-100	t,40-60	t,30-40	t,24-30	
Maximum ribosomal chain elongation rate:	12	16	18	20	21	amino acids/s
Maximum amount of ribosomes per cell:	6800	13500	26300	45100	72000	Ribosomes/cell
Protein per cell	100	156	234	340	450	Fg
Dry mass per cell (fg=10^{-15})	148	258	433	641	865	Fg
Average ribosome per mass	45,95	52,33	60,74	70,36	83,24	Ribosome/fg
Average maximum chain elongation:	551	837	1093	1407	1748	amino acids/s/fg

Reference:

Bremer H, Dennis P. Modulation of chemical composition and other parameters of the cell by growth rate. In Escherichia coli and Salmonella typhimurium. Neidhardt, F (ed) Washington, DC Am Soc Microbiol Press. 1996;(JANUARY 1996):1553.

Linear regression for elongation rate and protein fraction against growth rate

			Regression: RER vs Growth rate		
Growth rate (/h)	Max ribosomal elongation rate (RER) (molecule/s/fg)	in mmol/hr/gdw	slope	intercept	protein fraction (g/gdw)*
0,52	837,21	5,00	5,33	2,16	60,47%
0,83	1093,30	6,54	0,14	0,15	54,04%
1,19	1407,18	8,41	1,00	0,10	53,04%
1,54	1747,98	10,45	1535,29	2,00	52,02%

Therefore we have elongation rate = 5.33 x growth rate + 2.16 (mmol/hr/gdw)

*Protein fraction in the E. coli iAF1260 model was assumed to be 56.3%

Supporting Data - 5

Simulations with substrate worth 0.004 EUR (approximately 10 grams of substrate) are depictured in multiple simulations (0% to 100% sucrose faction of the substrate composition. Time is on the X axis, while different parameters are present in the Y axis for the different plot groups. The bold red line is the maximum productivity simulation.

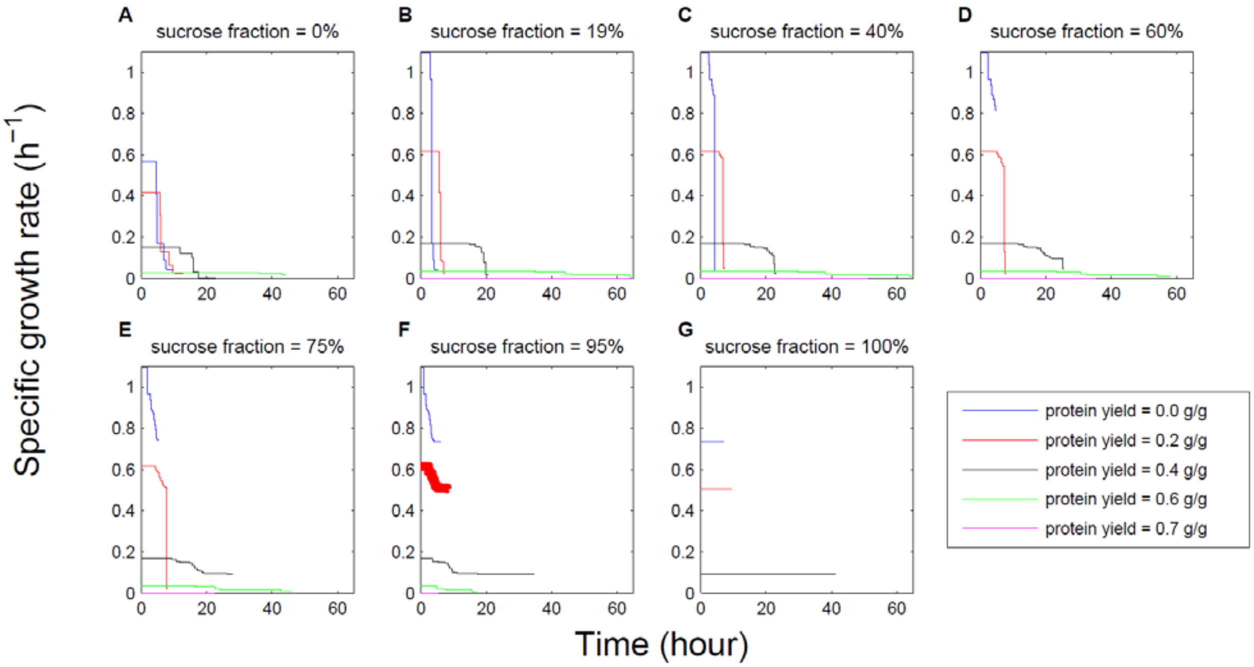

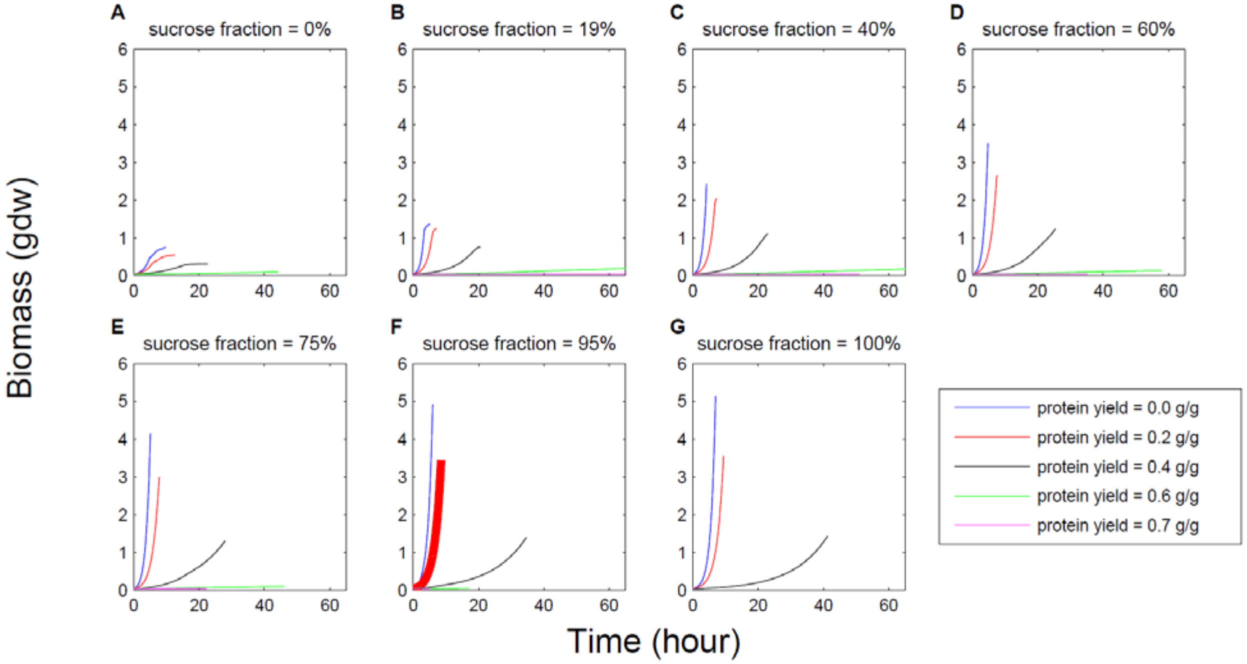

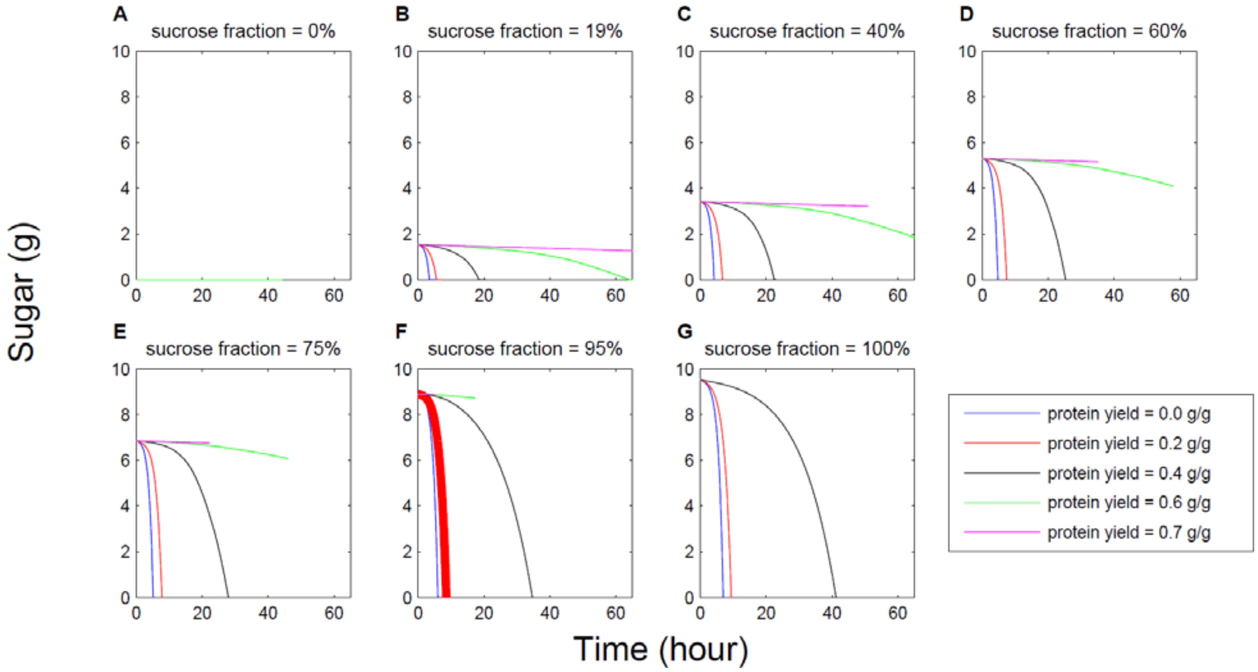
A
sucrose fraction = 0%
B
sucrose fraction = 19%
C
sucrose fraction = 40%
D
sucrose fraction = 60%
E
sucrose fraction = 75%
F
sucrose fraction = 95%
G
sucrose fraction = 100%
Sugar (g)
Time (hour)
protein yield = 0.0 g/g
protein yield = 0.2 g/g
protein yield = 0.4 g/g
protein yield = 0.6 g/g
protein yield = 0.7 g/g

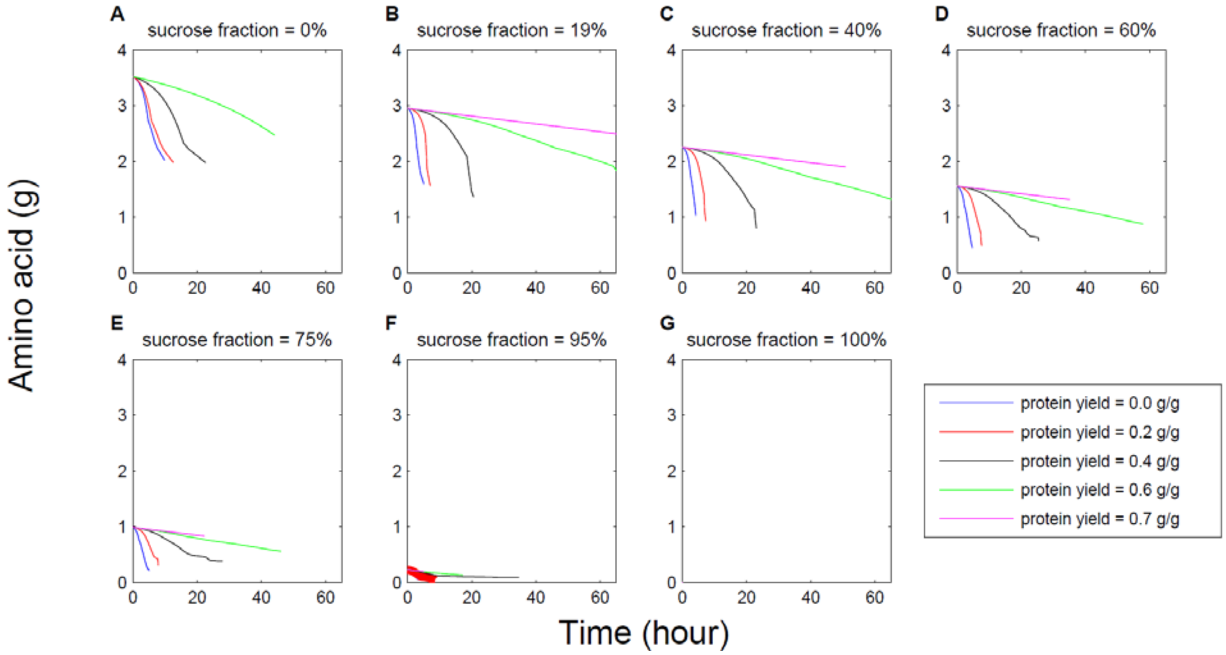
A
sucrose fraction = 0%
B
sucrose fraction = 19%
C
sucrose fraction = 40%
D
sucrose fraction = 60%
E
sucrose fraction = 75%
F
sucrose fraction = 95%
G
sucrose fraction = 100%
Amino acid (g)
Time (hour)
protein yield = 0.0 g/g
protein yield = 0.2 g/g
protein yield = 0.4 g/g
protein yield = 0.6 g/g
protein yield = 0.7 g/g

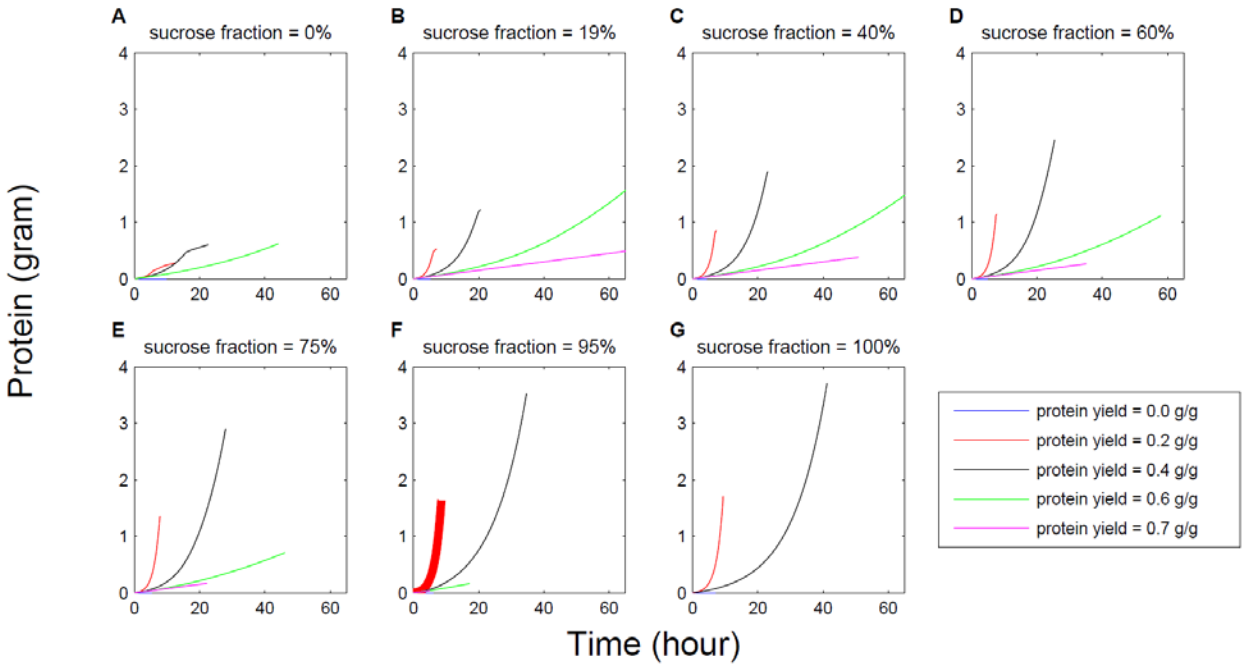
A
sucrose fraction = 0%
B
sucrose fraction = 19%
C
sucrose fraction = 40%
D
sucrose fraction = 60%
E
sucrose fraction = 75%
F
sucrose fraction = 95%
G
sucrose fraction = 100%
Protein (gram)
Time (hour)
protein yield = 0.0 g/g
protein yield = 0.2 g/g
protein yield = 0.4 g/g
protein yield = 0.6 g/g
protein yield = 0.7 g/g

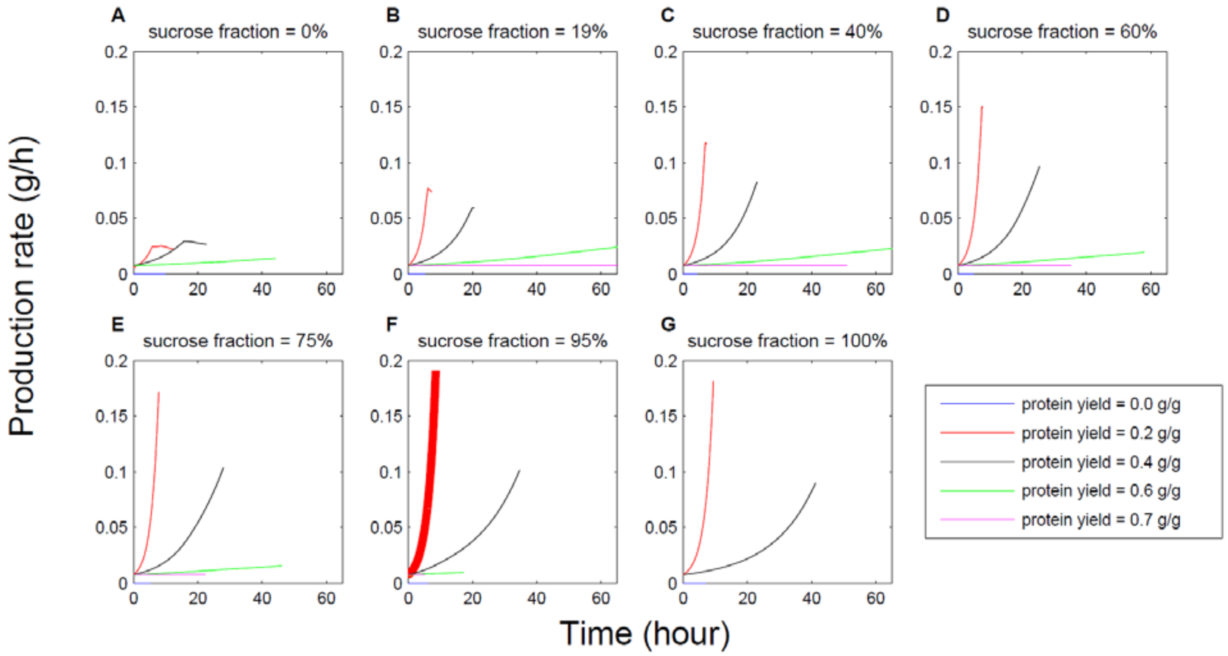
A
sucrose fraction = 0%
B
sucrose fraction = 19%
C
sucrose fraction = 40%
D
sucrose fraction = 60%
E
sucrose fraction = 75%
F
sucrose fraction = 95%
G
sucrose fraction = 100%
Production rate (g/h)
Time (hour)
protein yield = 0.0 g/g
protein yield = 0.2 g/g
protein yield = 0.4 g/g
protein yield = 0.6 g/g
protein yield = 0.7 g/g
